# Predictors of HIV status disclosure to sexual partners among People living with HIV/AIDS in Ethiopia

**Published:** 2012-12-28

**Authors:** Tadese Asfaw Erku, Berihun Megabiaw, Mamo Wubshet

**Affiliations:** 1 Epidemiology and Biostatistics Unit, College of Medicine and Health sciences, Debre Markos University, and Department of Psychiatry, School of Medicine, College of Health Science, Addis Ababa University, Ethiopia; 2 Department of Epidemiology and Biostatistics, Institute of Public Health, University of Gondar, Ethiopia; 3 Department of Environment Health, Institute of Public Health, University of Gondar, Ethiopia

**Keywords:** Disclosure, sexual partner, antiretroviral therapy, People living with HIV/AIDS, Ethiopia

## Abstract

**Introduction:**

Disclosure of HIV positive sero-status to sexual partners, friends or relatives is useful for prevention and care. Identifying factors associated with disclosure is a research priority as a high proportion of people living with HIV/AIDS never disclose in Ethiopia. This study was carried out to assess the magnitude and factors associated of HIV seropositive status disclosure to sexual partners among peoples living with HIV/AIDS.

**Methods:**

A hospital based cross-sectional study was conducted April-June, 2010, among systematically selected 334 HIV patients attending at Woldia hospital, Ethiopia. Data were collected through pre-tested questionnaire, using exit interview. Bivariate and multivariable logistic regression models were fitted to identify associated factors for disclosing their HIV seropostive status to sexual partner.

**Results:**

One hundred nineteen nine (59.6%) was females, 218(65%) was from urban area, 297(85.8%) are on antiretroviral therapy. The study found a significant association between higher educational status of the respondents (AOR:0.4; 95%CI (0.17-0.92)) and sexual partners (AOR: 9.0; 95% CI(2.8-29.3)), knowing HIV status of sexual partner (AOR:8.1; 95%CI(3.4 -19.2)), being on antiretroviral therapy (AOR:7.9; 95%CI(3.42-18.5)), having follow up counseling (AOR:5.26; 95%CI(2.2-12.5)), and being tested for HIV in ante natal care clinic (AOR:0.21; 95%CI(1.14- 6.46)) with disclosure of HIV status to sexual partner.

**Conclusion:**

The study concluded the need for giving more emphasis for the patients who are not on antiretroviral therapy and the need for giving emphasis on techniques how to disclose status to sexual partner.

## Introduction

Disclosure is a process, one that is positively linked to counseling, care and support. Disclosure of HIV status to sexual partners is an important prevention goal emphasized by the World Health Organization (WHO) and the Centers for Disease Control and Prevention (CDC) [1, 2]. Disclosure offers a number of important benefits to the infected individual and to the general public. In addition, HIV status disclosure may lead to improved access to HIV prevention and treatment programmes, increased opportunities for risk reduction and increased opportunities to plan for the future [3].

A person's ability to effectively prevent HIV transmission and acquisition is supported by knowledge of personal and partner HIV sero-status. Counseling and testing for HIV combined with disclosure of HIV sero-status to sexual partners can enable people living with HIV/AIDS (PLWHA) to seek appropriate care and treatment. Additionally, it can allow both PLWHA and uninfected persons to make informed choices about their sexual behavior [4].

To fuel the HIV/AIDS epidemic, HIV sero-positive individuals must interact unsafely with HIV-sero-negative individuals. Research indicates that, up to one third of individuals diagnosed with HIV infection continue to have unprotected sex, at times without informing their sexual partners, who may be of negative or unknown sero-status [2, 4]. Some public health interventions have focused on encouraging HIV sero-positive individuals to reveal their sero-status to their partners, predicated upon the assumption that disclosure will increase the safety of subsequent sexual activity with informed partners [5].

Despite public health benefits of disclosure, there are a number of potential risks, including loss of economic support, blame, abandonment, physical and emotional abuse, discrimination and disruption of family relationships. These risks may lead to choose not to share their HIV test results with their friends, family and sexual partners [6]. Report also showed that disclosure has significant impact on adherence to medical regimens [7].

However, there are few studies which document the extent of HIV sero status disclosure to sexual partner among PLWHA in Ethiopia. This study was carried out to assess magnitude and predictor of HIV sero-postive status disclosure PLWHA. We are confident that our study will provide baseline data for policy makers in developing appropriate evidence-based strategies to promote HIV sero positive disclosure to sexual partners among these individuals in Ethiopia.

## Methods

The study was conducted in Woldia hospital. It is found in Woldia town. The town is located 521 km north east of Addis Ababa, capital city of Ethiopia. In the hospital, medical, surgical, gynecology and obstetrics and pediatrics care, voluntary counseling and testing (VCT), ante retro viral therapy (ART) and treatment of opportunistic infection (TOI) services are available. Currently, 6135 PLWHA are utilizing the hospital routine services. The hospital is selected because it is one of the hospitals with large number of HIV patient flow in the region [8].

The sample size was calculated by using single population proportion formula. Assuming 69% of HIV positive individuals disclose their sero-positive status to sexual partners from similar study in Ethiopia [9], 95% level of confidence, taking 5% marginal error, 10% non response rate. Finally, 334 individuals were selected by systematic sampling method.

Data collection was conducted using pretested and structured questionnaire. The questionnaire was adopted from similar study [9, 10]. The questionnaire was translated from English to Amharic (Ethiopian national language) and then back translated to English to check the consistency. Finally the Amharic version was used for the data collection. In this study, individuals whose age ≤ 18 years and those who are acutely and seriously ill was excluded. Where as, individuals whose age ≥ 18 and who have sexual partner currently are included.

The questionnaire has six parts. These are; socio-demographic characteristics of the respondents and their sexual partner, health care related factors, disclosure experience, the reasons for disclosure and non disclosure of their HIV sero-positive status and perceived stigma and discrimination.

All the information where collected after taking oral informed consent from each respondents. Interview was carried out in strictly private room which is prepared near to ART clinic. Data was collected by 5 diploma nurse. Prior data collection, a one week compressive training was given for the supervisors and data collectors.

To ensure the quality of the data, daily meetings were held between the principal investigators and data collectors to troubleshoot problems that arose in the data collection process. In addition, inspection for completeness and quality of data collection was carried out daily by the supervisors and feedback was provided to data collectors. For the comfort of the respondents, data collectors of the same sex were applied.

The collected data were cleaned, checked, coded, entered and then analyzed by version 16 Statistical Package for Social Science (SPSS) statistical software. Bivariate analysis was carried out to see the crude effect of each factor. Multiple logistic regressions were computed. Variables that showed significant association in the bivariate analyses were fitted in to a multivariable logistic regression model to identify the independent contribution of each variable for HIV sero-positive status disclosure. Odds ratio with 95% confidence interval were calculated both to assess the association and measure the strength of the association between explanatory and outcome variables. P-Value less than 0.05 was taken as a cut off point to include the variable in to the model.

The information of the outcome variable, disclosure was recorded as Yes/No. where as income of the respondents was dichotomized as low and high income. In addition, the analysis of the educational status data was done by dichotomizing as illiterate “who con not read and write” and literate “who can read and write”. Each of the independent variable first run with the dependent variable to determine it's level of significance. Then only variable which are significantly associated with the outcome variable were fitted in the multiple logistic regression model to see the independent contribution.

Ethical clearance was obtained from University of Gondar, College of Medicine, and school of public health research ethical review board. A written permission was secured from Woldia Hospital medical director office. For eligible study participants, the purpose and the benefits of the study were discussed independently.

## Results

A total of 334 PLWHA were included in the study with the response rate of 100%. Majority of the respondents were from urban area 218(65%), 199 (59.6%) of them are females. The mean age was of the respondents were 30.5 ±4.5 years. Eighty four 84(25%) and 16(18%) of them are house wives and daily worker respectively. Majority 242(72%) of them are orthodox Christianity follower. About one third of (35.6%) of the respondents are primary school and the great majority of them 195(58.4%) earns < 500 ETB per month (Table 1).

**Table 1:** Socio-demographic characteristics of the respondents, Woldia Hospital, Ethiopia, 2010 (n=334)

Variables		Number	Percentage (%)
**Residence**	Urban	218	65.3
	Rural	116	34.7
**Sex**	Female	199	59.6
	Male	135	40.4
**occupation**	Farmer	97	29.0
	House wife	84	25.1
	Daily worker	60	18.0
	Merchant	46	13.8
	Governmental employee	43	12.9
	Other**	4	1.2
**Educational status**	Illiterate	111	33.2
	Read and write only	23	6.9
	Primary school	119	35.6
	Secondary school	44	13.2
	12 and above	37	11.1

** Driver

Majority 243(70.2%) were on ART and 297(85.8%) of the respondents are tested in VCT centers. Almost all of the study participants had taken pre test and post test counseling 331(99%), 306(91.6%) respectively.

In this study 78(23.3%) did not disclose their HIV status to sexual partners. Even though 256(76.7%) disclosed to sexual partners, 75(29%) of them is delayed disclosure. The Bivariate analysis showed that having more than 2 sexual partner in the last six months is 90% less likely to disclose than having one sexual partner (COR=0.069,95%CI (0.03,0.15)).

Concerning HIV risk sexual experience, 79 respondents have had unsafe sex the last 12 month. In this study, 36 (10.7%) are with HIV positive partners, 14(4.1%) are with HIV negative partners and 29 (8.6%) with unknown HIV status. From all respondents, 39(11.6%) of them reported more than two sexual partner in the last six month. In addition, PLWHA who have had unsafe sex with unknown HIV sero-status partner is 72% less likely to disclose than known one (COR=0.12, 95%CI (0.056, 0.286)).

The bivariate analysis showed that literate individuals (OR=0.56, 95%CI (0.35, 0.98)) and having more than two sexual partner (OR=0.069, 95%CI, (0.03, 0.15)) are less likely to disclose than others (Table 2). On top of this being on ART (COR 19.5, 95%CI (10.47, 36.65)), getting ongoing counseling service about disclosure (COR=3.8, 95%CI, (1.39,10.77)), tested for HIV in VCT centers (clinic) (COR=8.30,95%CI, 3.97,17.35) was significantly associated with HIV status disclosure to sexual partner.

**Table 2:** Variables which have significant association with disclosure of HIV seropositive status to sexual partner among people living with HIV/AIDS, Woldia Hospital, Ethiopia, 2010

Variables	Disclosed	Did not disclosed	COR (95%CI)	AOR(95%CI)
**Educational status of the respondent**				
Illiterate	95	39	1	
Literate	161	39	0.5(0.35-0.98)	0.4(0.17-0.92)
**Educational status of sexual partners**				
Illiterate	117	17	1	
Literate	139	61	1.67(1.0,2.7)	9.0(2.8,29.3)
**Knowledge of HIV status of sexual partner**				
Yes	231	26	18.4(9.8,34.5)	8.1(3.4,19.2)
No	25	52	1	
**Post test HIV care**				
Pre ART	33	58	1	
On ART	223	20	19.5 (10.47,36.65)	7.9(3.42,18.5)
**Ongoing Counseling**				
Yes	225	28	3.8(1.39,10.77)	5.26(2.2-12.5)
No	31	50	1	1
**Clinic where HIV testing was done**				
VCT	243	54	8.30(3.97,17.35)	1
ANC/PMTCT	13	24	1	0.21(0.04,0.94)
**Perceived stigma and discrimination**				
low	137	15	5.72(2.616,8.93)	2.27(1.14,6.46)
High	119	63	1	1

In this study, duration of follow up of HIV related service has significantly associated with disclosure. PLWHA who have had HIV related service follow up for more than 2 year are 2 times more likely to disclose than those who have follow up for less than 2 years (COR=2.48,95%CI(1.47,4.17)). On the other hand, there is an association between social related factors with disclosure to sexual partner. Being a member of HIV/AIDS association, seeing a person who discloses HIV status to the community, and perceived low stigma and discrimination was significantly associated with disclosure to sexual partner (Table 2).

After controlling multiple confounding factors by multiple logistic regressions, being literate is 60% less likely to disclose their sero-positive status to sexual partner than those who are illiterate (AOR=0.4, 95% (0.17, 0.92)). From all social related factors for disclosure of HIV status to sexual partners which were significant in bivariate analysis, only one variable, perceived stigma and discrimination, was significant after controlling for different confounding factors. Respondents who have low perceived stigma and discrimination is 2.27 times more likely to disclose to sexual partner than those who has high perceived stigma and discrimination (AOR=2.27,95%CI(1.14,6.46)).Similarly, individuals who knows their sexual partners HIV status is 8.1 times more likely to disclose than those who don't know their partner's HIV status (AOR=8.1,95%CI(3.4,19.2)).

The study showed that individuals who test for HIV in ANC or PMTCT clinic are 78% less likely to disclose than those who test in VCT clinics (AOR=0.228 95%CI, (0.08, 0.61)). Similarly those who get follow up care are 5.26 times more likely to disclose to sexual partner than those who didn't get (AOR 5.26,95%CI(2.2,12.5)). The multiple logistic regression output showed that respondents who are on ART are 7.9 times more likely to disclose than those who are on pre ART (AOR 7.9,95%CI(3.42,18.5)).

From a total of 256 individuals who disclose their HIV sero-positive status to sexual partner's and the reason listed by the participants for their disclosure was to get support from sexual partner and to benefit sexual partner to get medical care. On the other hand, the frequently listed reason by individuals who didn't disclose their HIV sero-positive status to sexual partner was perceived lack of communication skill to disclose (Table 3).

**Table 3:** Reasons for non disclosure of HIV sero-positive status to sexual partner among people living with HIV/AIDS, attending at Woldia hospital, Ethiopia, 2010, (n=78)

Reasons	Yes (%)	No (%)
Perceived lack of Communication skill	50(64.1)	28(33.9)
Fear of loss of confidentiality	45(57.6)	33(42.6)
Fear of abandonment	41(52.5)	29(47.5)
Fear of shaming family	27(34.6)	51(63.5)
hurt me physically	32(41)	46(69)
No enough time to discuss	13(16.6)	65(83.4)
Fear of accusation of infidelity	48(61.6)	30(38.4)
Not to worry him/her	30(38.4)	46(41.6)

## Discussion

Disclosure to sexual partners enables couples to make informed reproductive health choices that may ultimately lower the number of unintended pregnancies among HIV positive couples, and even reduce the risk of HIV transmission from mother to child.

In this study, the rate of disclosure to sexual partner is 76.6% and 29% of disclosure is delayed. The finding is lower than the research done in Hawasa referral hospital, Ethiopia, which is 85.7% and Jimma university hospital, Ethiopia, which is 90.8% of the respondents disclose to sexual partner [9, 10]. The reason could be the study subjects from Jimma and Hawasa university Hospitals may get adequate HIV related information easily than PLWHA in Woldia hospital, in which the hospital have poor infra structure and small number of health professional are working in.The other reason could be majority of the respondents are females which tends to decrease the disclosure rate [11]. Regarding to delayed disclosure, the result is comparable to different study done Ethiopia, this could be because of individual may stay up to the disease becomes sever and physical signs and symptoms are pronounced that enforce them to disclose.

**Figure 1:**
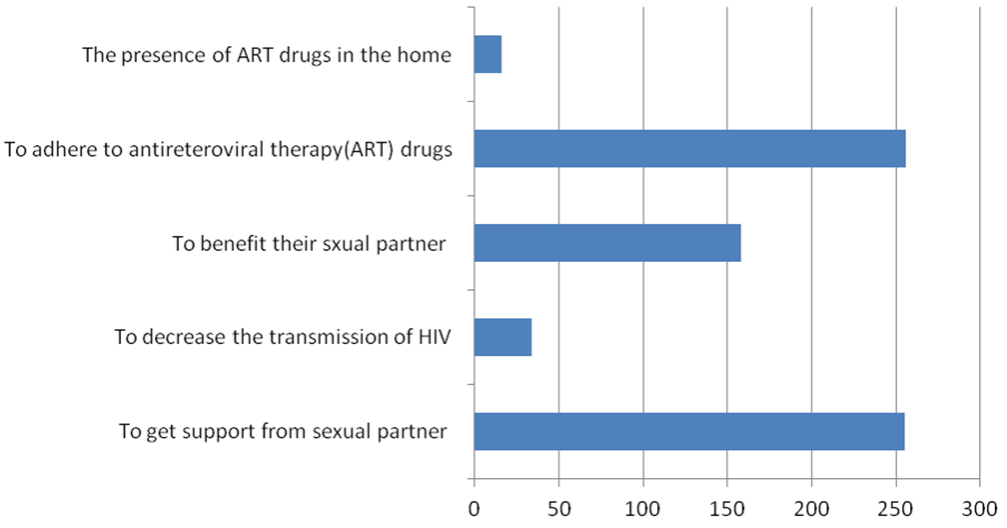
Reasons for their disclosure of seropositive HIV status listed by the respondents of HIV status to sexual partner among PLWHA attending at Woldia Hospital, Ethiopia, 2010, (n=256)

In this study the reason for their nondisclosure of HIV sero-positive status to sexual partners was perceived lack of Communication skill, fear of loss of confidentiality fear of accusation of infidelity, fear of abandonment. This reasons were similar to studies done in different developing countries like Uganda and south Africa, Tanzania and Kenya on PLWHA [12–14] and south east Ethiopia as well [10]. This could be because of most of the majority participates are illiterate and might not get comprehensive information regarding the importance disclosure in the VCT or ANC clinics and the fact that in different rural developing countries including Ethiopia, males are the source of income for the household than females, that might enforce the to conceal their sero-positive status (Figure 1).

As the study showed, having unsafe sex in the last 12 month was significantly associated having HIV negative or unknown serostatus. The finding is in line with a survey done South African that the 42% of participants have had unsexes without disclosure [8]. Condom utilization by the respondents were 83.8% and only 67.9% use condom all the times during sexual intercourse. This finding is also supported by a case study done in Brazil on PLWHA that, there is a relationship between lack of HIV disclosure and unsafe sex behavior among [10]. This may put the couple at risk of infecting by the virus for those who are HIV negative and re infection by new strains and developing resistance.

Interestingly, there is a relation ship between ART initiation and disclosure of HIV status to sexual partner. The finding is inline with the study done in Tanzania and Mozambique disclose their HIV test result to sexual partner after 12 month of Initiation of HAART [16]. This could be primary PLWHA on ART often reported feeling of comfortable with their status. As a result of overcoming internalized feelings of shame facilitated disclosure of HIV status. Secondly, Interaction of this groups with other HIV infected people and see another infected person disclosed his or her status tended to gives PLWHA more courage to disclose their status to sexual partner. Thirdly, in Ethiopia, ART is initiated after clinical AIDS signs and symptoms occurred in which PLWHA cannot easily be concealed (hide) their HIV status from the community.

The study finding shows that PLWHA with high perceived stigma and discrimination is one of the independent factors for HIV status disclosure to sexual partner. This finding is similar to the result of different researches in different African countries [17] that as stigma and discrimination very high, leading to a lower rate of disclosure, because people do not see the reason to disclose. This could be because of disclosing of HIV status is a risk and burden for the individuals apart from the physical and emotional consequence of the disease.

In this study, the place where HIV testing and counseling was carried out has significant relationship with disclosing sero-positive status to sexual partner that being tested in ANC or PMTCT was less likely to disclose than those who are tested in VCT clinic. This finding is similar to a research done in Jimma and Hawassa referral Hospital [9, 18]. The reason might be because individuals tested in VCT clinics are think of about HIV risk behaviors and they came for testing after a long term consultation with significant others than individuals tested in ANC clinics that are tested for HIV probably by being pregnant or during child birth.

This study come up with not knowing the HIV status of sexual partner is significantly associated with less likely to disclose which is inline with a case control study done in Mityana, Uganda [14]. This might be knowing HIV status of sexual partner gives strength and gives courage to disclose their own HIV status. In addition a person's ability to effectively prevent HIV transmission and acquisition is supported by knowledge of personal and partner HIV sero-status.

Although the interviews were carried out in strictly private, the possibility of social desirability bias cannot be totally eliminated as the study touches very sensitive issues. In general this study came up with findings which have a policy implication and need to give more emphasis in the VCT clinics and for those who are not on ART, so as to decrease the incidence of the disease.

## Conclusion

Disclosure of HIV sero positive status to sexual partner is promising. But one third of the disclosure is delayed disclosure. The major reasons for their nondisclosure of HIV status were perceived lack of communication skill, fear of loss of confidentiality, fear of abandonment and fear of accusation of infidelity. In addition non disclosure of HIV status was high among individuals who are not on antiretroviral treatment and having test in VCT clinics. Further perceived stigma and discrimination is still a bottle neck for disclosure of sero positive status.
